# Ancient Mitochondrial Genomes Reveal the Absence of Maternal Kinship in the Burials of Çatalhöyük People and Their Genetic Affinities

**DOI:** 10.3390/genes10030207

**Published:** 2019-03-11

**Authors:** Maciej Chyleński, Edvard Ehler, Mehmet Somel, Reyhan Yaka, Maja Krzewińska, Mirosława Dabert, Anna Juras, Arkadiusz Marciniak

**Affiliations:** 1 Institute of Archaeology, Faculty of Historical Studies, Adam Mickiewicz University in Poznań, Umultowska 89D, 61-614 Poznań, Poland; arekmar@amu.edu.pl; 2 Department of Biology and Environmental Studies, Faculty of Education, Charles University, Magdalény Rettigové 4, 116 39 Prague, Czech Republic; edvard.ehler@pedf.cuni.cz; 3 Department of Biological Sciences, Middle East Technical University, 06800 Ankara, Turkey; somel.mehmet@googlemail.com (M.S.); yakaryhn@gmail.com (R.Y.); 4 Archaeological Research Laboratory, Department of Archaeology and Classical Studies, Stockholm University, Lilla Frescativägen 7, SE-106 91 Stockholm, Sweden; maja.krzewinska@arklab.su.se; 5 Molecular Biology Techniques Laboratory, Faculty of Biology, Adam Mickiewicz University in Poznan, Umultowska 89, 61-614 Poznań, Poland; mirkad@amu.edu.pl; 6 Department of Human Evolutionary Biology, Institute of Anthropology, Faculty of Biology, Adam Mickiewicz University in Poznań, Umultowska 89, 61-614 Poznań, Poland; annaj@amu.edu.pl

**Keywords:** ancient DNA, Neolithic, kinship

## Abstract

Çatalhöyük is one of the most widely recognized and extensively researched Neolithic settlements. The site has been used to discuss a wide range of aspects associated with the spread of the Neolithic lifestyle and the social organization of Neolithic societies. Here, we address both topics using newly generated mitochondrial genomes, obtained by direct sequencing and capture-based enrichment of genomic libraries, for a group of individuals buried under a cluster of neighboring houses from the classical layer of the site’s occupation. Our data suggests a lack of maternal kinship between individuals interred under the floors of Çatalhöyük buildings. The findings could potentially be explained either by a high variability of maternal lineages within a larger kin group, or alternatively, an intentional selection of individuals for burial based on factors other than biological kinship. Our population analyses shows that Neolithic Central Anatolian groups, including Çatalhöyük, share the closest affinity with the population from the Marmara Region and are, in contrast, set further apart from the Levantine populations. Our findings support the hypothesis about the emergence and the direction of spread of the Neolithic within Anatolian Peninsula and beyond, emphasizing a significant role of Central Anatolia in this process.

## 1. Introduction

Neolithic Çatalhöyük (7100–5950 BC) is a world-renowned Neolithic settlement. Its size, remarkable preservation, presence of numerous works of Neolithic art, and large amounts of archeological data obtained through meticulous excavation have consolidated its unquestioned importance in the identification of a wide range of constituent elements of the Neolithic [1]. The settlement was composed of a conglomeration of clustered neighborhoods with clearly defined modular house units [2]. All houses were apparently occupied and used for domestic purposes [3]. Burials were located under the floors of most buildings, especially under elevated platforms in northern and eastern parts of the living rooms. However, some of the buildings, notably the ones with more elaborate art installations, contained more burials (up to almost 70 individuals, more than one would expect from the estimated number of their inhabitants), implying their special status [4]. Those buildings are thought to have been “history houses” that provided or controlled ancestors and rituals for a larger kin or other group [3].

Initially it was proposed that Çatalhöyük individuals buried together in the same building were biologically related, and groupings of houses and constituting neighborhoods were defined by biological affinity [5]. Then, an uneven distribution of burials among different houses was interpreted as evidence for some of them being a burial place for larger household communities, composed of a number of houses inhabited by nuclear families [6]. A recent study based on dental phenotypes of individuals found in Çatalhöyük burials showed that individuals with close biological affinity spanned across several buildings [7]. This result was interpreted as evidence of the lack of kinship patterning in burials found at the site. However, the correlation between biological distances based on both morphological traits and genetic kinship is poorly understood, as both types of data are rarely available for the same set of samples. In the few studies where direct comparison between morphological and genetic data was available, the results were inconsistent, pointing towards weak correlation [8]. Several approaches and tools for genetic kinship estimation based on ancient DNA have been recently published [9,10,11], and although these tools were developed with low coverage data in mind, they still depend on significant overlap in nuclear single nucleotide polymorphisms (SNPs) between analyzed samples. However, where overall ancient DNA (aDNA) preservation between samples is poor and/or deeper sequencing data is not feasible, mitochondrial (mt) genomes can be used to exclude maternal kinship [12].

The emergence and expansion of the Neolithic within and outside of Anatolia is another issue that could be addressed with ancient DNA data from Çatalhöyük. This process is thought to be a sum of several waves and trajectories of migration [13,14,15]. The Neolithic in Central and South-western Anatolia is thought to have developed under the influences from the upper Euphrates valley in the span of a thousand years, with the earliest evidence of some degree of Neolithic lifestyle seen in Central Anatolia in the second half of the 9th millennium BC [16]. However, the emergence of Neolithic societies in Central Anatolia was also proposed to be an autonomous process, involving local hunter-gatherers adopting the Neolithic lifestyle under the influence of farming communities from South-eastern Anatolia and Levant [16,17,18]. Furthermore, it has additionally been proposed that the region of Central Anatolia might not have contributed significantly to the subsequent westward movement of Neolithic tradition, as both archeological [19,20] and zooarchaeological [21] data suggest that it constituted a distinctive cultural zone. At the same time, the maritime colonization originating in the Levantine coast has been proposed as the major factor contributing to the development and the spread of the Neolithic in the Aegean coast of western Anatolia [22]. It is thought that this process did not involve any local populations, as Mesolithic occupation was sparse in the parts of the region where the Neolithic first appeared [14].

Çatalhöyük was undoubtedly a part of a large, far-reaching exchange network [23,24] and could have potentially participated in the exchange of both goods and ideas. Elements of Çatalhöyük origin began to emerge in particular in North-western Anatolia in the middle of the 7th millennium BCE [25]. This process was unquestionably complex, presumably involving several subsequent impulses [15], as it took two millennia for the Neolithic to spread first to western Anatolia, with the earliest dates for the Neolithic being around the late 8th millennium BC [14], and then towards Marmara Region in the late 7th millennium BC [26]. The questions concerning to what extent those impulses towards west and North-western Anatolia were connected with gene flow and what was the role of the autochthonous hunter-gatherers in the spread are yet to be resolved. In some parts of the region intermediate and mixed traditions and economies have been observed [27]. At the same time, genomic data, both from the Marmara Region [28,29] and Central Anatolia [30], shows the genetic similarity of those regions and their close genetic affinity with Central European Neolithic populations. Those results support the leading role of the terrestrial route of the Neolithic spread both within and outside of the Anatolia.

In this work we address both the question of maternal kinship relations of individuals from Çatalhöyük, and the genetic affinities of Central Anatolian populations and what follows their potential relation to the westward spread of the Neolithic. We present ten new complete mitochondrial genomes from Çatalhöyük individuals buried under the floors of adjacent buildings dated to classic levels (Mellaart Phase VI A) of its occupation.

## 2. Materials and Methods

**Figure 1:**
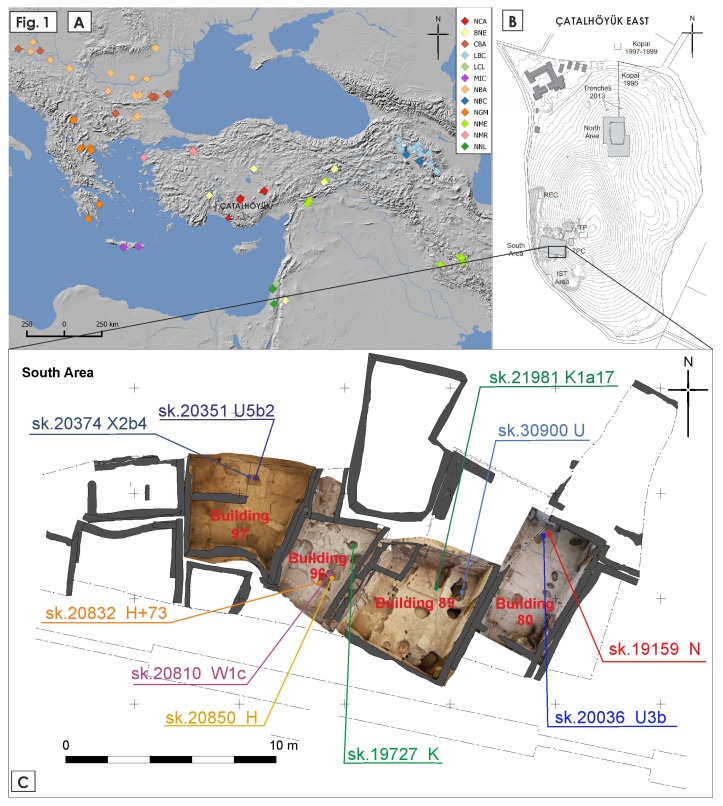
(**A**) The location of Çatalhöyük and other sites with complete mitochondrial genomes used as the reference for the study: (BNE) Bronze Age Near East, (CBA) Chalcolithic Balkans, (LBC) Late Bronze Age Caucasus, (LCL) Late Chalcolithic Levant, (MIC) Minoan Create, (NBA) Neolithic Balkans, (NBC) Neolithic to Bronze Age Caucasus, (NCA) Neolithic Central Anatolia, (NGM) Neolithic Greece and Macedonia, (NME) Neolithic Middle East, (NMR) Neolithic Marmara Region, (NNL) Natufian and Neolithic Levant. (**B**) Outline of the Çatalhöyük East mound with visible excavation areas. (**C**) Close-up of the excavation area and buildings targeted for the study with the locations and the obtained mitochondrial haplogroups of the individuals reported in the paper.

Four adjacent, roughly contemporary buildings from Çatalhöyük South Area, dated to Mellaart Phase VI A (6450–6380 cal. BC [31]), were selected for the study (Figure 1C). We assumed that the selected buildings represented ordinary houses as neither of them was recognized as a “history house” by the researchers of the site, however, an above-average number of art installations were found in building 80, and all of the buildings, with the exception of building 89, contained more than 10 burials. All available individuals excavated from those buildings were sampled. Where possible, petrous part of temporal bones were collected. The samples were all taken from the Çatalhöyük Research Project depot with the use of disposable gloves and facemasks. In total, 47 bone samples were acquired from 37 skeletons, including ten individuals from building 96, six from building 97, five from building 89, and 16 from building 80. The detailed information on the selected samples can be found in the Supplementary Information Text and Supplementary dataset S1.

DNA was extracted from teeth and petrous parts of temporal bones in laboratories dedicated to working with human aDNA. The surface of the samples was decontaminated with the use of 2% bleach and UV light, and only the inner part of both the teeth and petrous bones were drilled for extraction. DNA isolation was performed both at the Middle East Technical University in Ankara (METU), Turkey, and at the Faculty of Biology, Adam Mickiewicz University in Poznan (AMU), Poland. In the METU laboratory, the DNA was extracted using a silica-powder-based method and modified binding buffer, as described by Allentoft et al. [32]. In the AMU facility, the DNA was extracted using a silica-based method as in [33], with modification by [34]. Total genomic DNA libraries for all samples were constructed at the AMU laboratory using the methods described previously by Juras et al. [12].

The genomic libraries underwent Illumina sequencing (150 bp PE, ca. 1.4 mln reds/sample) at the Science for Life Laboratory (SciLifeLab) facility in Stockholm (NGI Stockholm), Sweden. All preliminary pipeline computations of the sequencing data were undertaken on resources provided by the Swedish National Infrastructure for Computing (SNIC) through the Uppsala Multidisciplinary Center for Advanced Computational Science (UPPMAX) [35].

The RNA baits for capture enrichment of complete mitochondrial genomes were prepared following the protocols described in Juras et al. [36]. Two rounds of mitochondrial DNA (mtDNA) enrichment were carried out on 22 libraries that showed either a proportion of reads mapping to human reference genome (version hs37d5) equal to at least 0.4%, or mtDNA coverage ranging from 0.02 × to 5 × after initial Illumina shotgun screening (Supplementary dataset S1). The libraries enriched in mtDNA were then sequenced on Illumina HiSeq2500 (125 bp, paired end, each library 1/80 lane) in the SciLifeLab facility in Stockholm (NGI Stockholm), Sweden.

DNA sequencing data from both shotgun screening and mtDNA capture were processed with the use of a customizable analytical pipeline, described in [37]. The adapters were removed and read pairs were merged, requiring an overlap of 11 bp and summing up base qualities using MergeReads-FastQ_cc.py script, according to Meyer & Kircher [38]. BWA software package version 0.7.8 [39] with the parameters -n 0.01 -o 2 and disabled seeding, was used to map merged reads as single-end reads against both the revised Cambridge Reference Sequence (rCRS) [40,41] (GenBank: NC_012920) and human reference genome (version hs37d5). To collapse duplicate sequence reads with identical start and end coordinates, FilterUniqueSAMCons.py was used [38]. The ratio of reads mapping to Y and X chromosomes (Ry) (with mapping quality greater than 30) was calculated to assign molecular sex of individuals [42].

The mtDNA capture binary alignment map (bam) files were merged with shotgun screening data, using the merge tool from SAMtools package v1.8 [43]. Misincorporation patterns for merged files were assessed using mapDamage v2.0.5 with the default parameters [44]. Contamination estimates for mtDNA sequences were then preformed using both contamMix_1.0-10 script [45] and Schmutzi package v1.5.4 [46] with the default parameters. Any sample that failed at least one of those tests, showing more than 18% contaminating sequences, was discarded from further analysis. Consensus sequences were built using ANGSD v0.910 [47]. Only reads with a mapping score of 30, a minimum base quality of 20, and positions with a minimum coverage of 3 were accepted [48]. All the computations were performed using resources provided by The Polish Grid Infrastructure (Pl-Grid). Mitochondrial haplogroups (hgs) were assigned for each individual utilizing HAPLOFIND [49], and Haplogrep [50] both based on the PhyloTree phylogenetic tree build 17 [51]. Biomatters IGV software v2.3.66 [52] was used to visualize final sequences, as well as mutations reported as unexpected or missing in the original binary alignment map (BAM).

For comparative analyses, complete ancient mtDNA genomes were obtained from the literature and the ancient human mitochondrial genomes database (AmtDB) [53]. Where available reconstructed fasta files were acquired, and in cases where only whole genome data was available, the mitochondrial genomes were reconstructed from the available bam files using the pipeline described above. All samples were then grouped into sets of at least 10 individuals based on their dating, geographical location, and/or attribution to particular archeological cultures. Only one sample from the confirmed kin pairs and groups was selected for population analyses. Additionally, READ [10] was used on 67 reference Neolithic Anatolian and Near Eastern samples in order to detect potential genetic kinship relations missed in previous studies. All comparative populations and samples used for principal component analysis (PCA), t-Distributed Stochastic Neighbor Embedding (t-SNE), and pairwise genetic distances (F_ST_) are described in detail in Supplementary dataset S2.

For the purpose of this study, due to the limited number of samples available for Iran and Turkmenistan Neolithic and Chalcolithic, the samples were grouped together into Neolithic Middle East group (NME). Similarly, Pre-Pottery Neolithic samples from Jordan were grouped together with epipaleolithic Natufian samples from the same region into the Natufian and Neolithic Levant group (NNL). Furthermore, the Bronze Age samples from Turkey were grouped together as Bronze Age Near East group (BNE) with Jordan samples from the same period, and Neolithic, Chalcolithic, and Early Bronze Age samples from Armenia were merged into the Neolithic to Bronze Age Caucasus group (NBC).

The Çatalhöyük samples were analyzed as part of the Central Anatolia Neolithic group containing additionally three individuals from Boncuklu Höyük and three from Tepecik-Çiftlik sites. It has been shown that the European gene pool was shaped by three major ancestral populations, including autochthonous hunter-gatherers, and two migrant groups from Near East in the Early Neolithic and Eurasian steppe in the Late Neolithic period [54,55,56]. However, since this work is mostly focused on genetic relationships within the early farming populations, all the analyses were performed using both: (i) only the Neolithic to Bronze Age populations from Near and Middle East with the addition of initial farming populations from Europe (as seen on (Figure 1A)), and (ii) all the above with the addition of the Yamnaya steppe groups and hunter-gatherers from Europe (divided into Western, Eastern, and Balkan populations), added as proxies of the other two major components of the European gene pool. Only one individual in each pair of known first degree relatives was used in the analyses. Additionally, individuals for which less than 85% of the mitochondrial genome was recovered were excluded from the analyses.

The map in Figure 1 was generated using QGIS 2.12.2. PCA of mtDNA hgs frequencies was calculated using Python 3.5 and the Scikit-learn v. 0.18.1 [57] package. The PCA results and mtDNA hgs loadings were plotted with the use of Matplotlib 1.5.1 Python package [58].

A centroid-based clustering approach was used to examine the PCA results to search for logical clusters within our data. The k-means method was used, as implemented in Scikit-learn v. 0.18.1 Python package [57], to the first five principal components of the PCA analysis. To further explore the relatedness of populations according to the mtDNA hgs frequencies, we ran the t-SNE analysis [59] as implemented in the Scikit-learn (18.1) Python package. F_ST_ values were computed in Arlequin 3.5 [60] on the same sets of samples, excluding those with less than 85% of mitochondrial genome reconstructed, using Nei’s average number of pairwise differences [61] and 10,000 permutations to estimate the *p*-values. To visualize F_ST_ values, multidimensional scaling (MDS) analysis was employed, using the Python Scikit-learn 0.18.1 package [57].

## 3. Results

We found the overall DNA preservation on the site to be rather poor, as a majority of the samples had less than 1% of endogenous human DNA, with notable exceptions of sk. 21981 (Table 1 and Supplementary dataset S1). Since only one sample yielded enough data to reconstruct the mitochondrial genome based solely on the results of shotgun sequencing, hybridization-based enrichment in mtDNA was therefore performed in the remaining cases. After capture, nine more complete mitochondrial genomes were obtained for the samples that passed both authenticity tests and displayed damage patterns typical for aDNA, including C-T and G-A transitions at the 5′ and 3′ ends of DNA fragments, respectively (Supplementary Figure S1). Molecular sex was assigned in the case of seven individuals, for which at least 800 reads were mapped to sex chromosomes in the shotgun screening (Supplementary Figure S2). In one case, the result matched the sex assigned based on the morphology, and in the remaining six cases the morphological sex was not available due to the low biological age of the individuals. A total of three individuals, including two children, were found to be females, and five more children were determined to be males (Table 1).

The mitochondrial genomes were obtained for four individuals buried under the floors of building 96, two individuals from building 97, and two from buildings 89 and 80 (Figure 1C). All individuals, for which we have reconstructed mitochondrial genomes, were assigned to different mtDNA lineages, present in ancient neighboring Neolithic and Chalcolithic populations and common among modern-day Eurasian populations (Table 1). Three individuals were assigned to U lineage (haplogroups U, U3b, U5b2), two to K lineage (K and K1a17), two to H lineage (H and H+37), and the three remaining individuals were assigned to X, N, and W lineages. The mitochondrial genomes were deposited in GenBank under accession numbers MK308698-MK308707.

Additionally, to the individuals excluded from PCA and t-SNE analyzes based on previous reported genetic kinship, two more were found to belong to pairs of first-degree relatives based on the READ analysis (Supplementary Figure S3 and Supplementary Table S1). Both pairs were found in other Neolithic Central Anatolian sites in Boncuklu Höyük and Tepecik-Çiftlik.

The results of PCA and t-SNE analyses show that the Neolithic Central Anatolian population falls within variations of other Neolithic and Chalcolithic populations from both the Middle and Near East, and Neolithic and Chalcolithic populations from Europe. In contrast, all those groups are set apart from both steppe and hunter-gatherer populations, to the point where the highest average silhouette for the k-means clustering of frequency-based PCA results for all the populations is for k value of 2, forming two clusters: steppe plus hunter-gatherer and other populations (Supplementary Figure S4).

**Figure 2:**
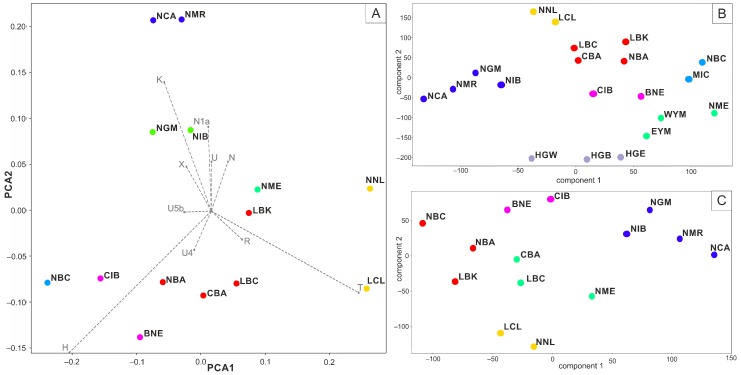
(**A**) Principal component analysis (PCA) plot with populations clustered according to k-means clustering (k = 7). (**B**,**C**) Plots of frequency based t-Distributed Stochastic Neighbor Embedding (t-SNE) with extended and reduced sets of populations clustered according to k-means clustering (k = 7 and k = 5, respectively). (BNE) Bronze Age Near East, (CBA) Chalcolithic Balkans, (LBC) Late Bronze Age Caucasus, (LCL) Late Chalcolithic Levant, (MIC) Minoan Create, (NBA) Neolithic Balkans, (NBC) Neolithic to Bronze Age Caucasus, (NCA) Neolithic Central Anatolia, (NGM) Neolithic Greece and Macedonia, (NME) Neolithic Middle East, (NMR) Neolithic Marmara Region, (NNL) Natufian and Neolithic Levant, (CIB) Chalcolithic Iberia, (NIB) Neolithic Iberia, (LBK) Linear Pottery Culture, (WYM) Western Yamnaya, (EYM) Eastern Yamnaya, (HGW) hunter Gatherers West, (HGE) Hunter Gatherers East, (HGB) Hunter Gatherers Balkans.

With the outliers excluded, the Neolithic Central Anatolian (NCA) population is always shown to be closely related to the Neolithic Population from the Marmara region (NMR) belonging to the same cluster in both PCA (Figure 2A) and t-SNE (Figure 2B,C) based analyses. The two form their own cluster in the PCA plot of the two first variables, describing 52.07% of variance and shown combined with the k-means clustering (with the k value of 7 as the best representation of the data, with the average silhouette of 0.3141) (Figure 2A) are far apart from Levantine populations, both Neolithic (NNL) and Chalcolithic (LCL). Other k-means variants (from 2 to 8) can be found in Supplementary Figure S4.

Additionally, those two groups, NCA and NMR, cluster together with Neolithic Greece and Macedonia (NGM) and Neolithic Iberian (NIB) samples in k-means clustering of t-SNE results, when analyzed with both the narrowed and extended datasets (k value of 7, average silhouette 0.5067 and k value of 5 average silhouette 0.421, respectively (Figure 2B,C—blue cluster)). While the t-SNE plots places Levantine populations (NNL and LCL) closer to Anatolian populations than PCA, they still tend to form their own cluster in k-means, clustering starting with the k value of 5 (Supplementary Figure S4C,D).

**Figure 3:**
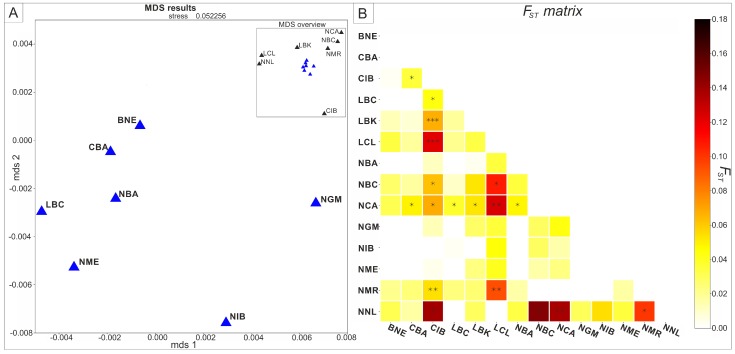
(**A**) Multidimensional scaling (MDS) plot of pairwise genetic distances (F_ST_) values obtained for complete mitochondrial genomes, (**B**) Matrix of F_ST_ values, defined by color, statistical significance *p* < 0.05 (*), *p* < 0.005 (**), *p* < 0.0005 (***). (BNE) Bronze Age Near East, (CBA) Chalcolithic Balkans, (LBC) Late Bronze Age Caucasus, (LCL) Late Chalcolithic Levant, (MIC) Minoan Create, (NBA) Neolithic Balkans, (NBC) Neolithic to Bronze Age Caucasus, (NCA) Neolithic Central Anatolia, (NGM) Neolithic Greece and Macedonia, (NME) Neolithic Middle East, (NMR) Neolithic Marmara Region, (NNL) Natufian and Neolithic Levant, (CIB) Chalcolithic Iberia, (NIB) Neolithic Iberia, (LBK) Linear Pottery Culture.

When looking at the F_ST_ values (Figure 3B and Supplementary Table S3) based on the complete mitochondrial genome sequences and MDS plot of these values (Figure 3A), the Neolithic Central Anatolia is again shown to be statistically significantly set apart from Levantine populations, (F_ST_ = 0.13737, *p* = 0.00446 for NNL and F_ST_ = 0.12420, *p* = 0.00059 for LCL). The NCA is also closely, but not statistically significantly, related to the population from the Marmara Region (F_ST_ = 0.01032, *p* = 0.64033) and the Neolithic to Early Bronze Age samples from Caucasus (NBC) (F_ST_ = 0.01074, *p* = 0.61340).

## 4. Discussion

*Maternal kinship and social structure of Çatalhöyük*

Our results show that all ten obtained genomes belong to different mitochondrial haplotypes. The case of the individuals buried within building 96 is especially interesting, as four different mitochondrial haplotypes suggest that at least four different maternal lineages were present in the group of individuals interred within this particular house. Such a high variability of mitochondrial haplogroups in a kin group, especially among children and females, could be explained by patrilocality. Assuming the house, as a typical Çatalhöyük structure, was occupied for 3–4 generations [62] and inhabited by a matrilocal or bilateral biological kin group, the chances of finding four individuals representing different maternal lineages and matching the sex and age of the individuals is implausible. However, to support this interpretation, either the paternal lineages reflected in Y chromosome data should be analyzed, or a precise estimate of the size of the kin group in question is needed. As building 96 has not been excavated in its entirety [63], only ten individuals have been unearthed to date. One cannot rule out the possibility that more deceased are still be found beneath its floor. As the number of individuals buried within a single house in Çatalhöyük varies from several to around 70 [3,4], it is difficult to reliably estimate how many individuals might have been buried in building 96. Any interpretation based solely on the data presented here would not be strongly justified, however, the lack of biological kinship between Çatalhöyük burials within a single house was also proposed based on metric and non-metric morphological dental traits [7]. The authors of this study further noted the absence of a distinctive correlation between obtained biological distances and spatial distribution of burials within and between buildings. This led scholars to propose an alternative hypothesis, suggesting that biological kinship was not the main factor used when selecting the individuals to be interred in a particular building. Southwest Pueblo societies from Northern America are often used as the best-fitting ethnographic reference of how such non-kin groups could have been organized [64]. Several analogies with Çatalhöyük are seen, especially in the Western Pueblo groups, where social units of the house intersect with small-scale religious sodalities. Such systems are characterized by ritual-based social organization, where biological kinship is secondary to alternative networks of affiliation [64]. Those models could potentially explain the variability in both genetic and morphological data, together with the uneven distribution of number of burials found within different buildings in Çatalhöyük.

However, the neighborhood was also proposed as a basic unit of both spatial and social organization of Çatalhöyük [3,6]. In this interpretation, a cluster of houses confined by refuse areas would represent a social unit, such as an extended family, and the burial location within the neighborhood would be defined by various factors, such as age or social status. Richly decorated central houses of those clusters with an excess of individuals interred under their floors would hold a special role in those neighborhoods, and are defined as history houses [3]. The group of individuals inhabiting such a neighborhood, in addition to the extended family, could also include non-related individuals [65]. The buildings studied here, as they are all adjacent to each other, and not separated by any potential borders, could all belong to one neighborhood. In the light of the aforementioned hypothesis, the group of individuals buried under one house could include members of much larger kin-groups than just 3–4 generations of a nuclear family and therefore the likelihood of them not sharing mitochondrial haplotype and still being biologically related would be higher.

On the other hand, direct kinship suggested by mitochondrial haplotypes and confirmed in the course of this study with the use of the READ method (Supplementary Figure S3 and Supplementary Table S1), was found in the individuals buried within or around the same structures, both in Tepecik-Çiftlik and Boncuklu Höyük. The latter site has been suggested to be one of Çatalhöyük’s possible genetic predecessors [18]. This hypothesis is based on its close proximity to Çatalhöyük, its occupation ending before Çatalhöyük was established, and numerous parallels in customs and rituals between those two sites noted by some researchers [16,17]. The direct cultural parallels between Çatalhöyük and Tepecik-Çiftlik are less prominent, as both elements of Central Anatolian and Mesopotamian tradition are present in its earlier levels, from which the discussed burials come from [66].

Although only mitochondrial genomes were obtained, when compared with available morphological [7] and spatial data [6], the results support the notion that burials found within Çatalhöyük buildings belonged either to large patrilocal or non-kin groups. To further explore what role the biological kinship played in the selection process of burial places in Çatalhöyük, nuclear data is needed to estimate further degrees of relatedness within larger groups, representing a substantial fraction of individuals buried within at least one building in Çatalhöyük.

*The genetic affinities of Central Anatolian Neolithic populations*

The genetic affinities of the Çatalhöyük inhabitants, pooled together with other Central Anatolia Neolithic individuals, unsurprisingly shows that this group was closely related to Near and Middle Eastern Neolithic and Chalcolithic populations, especially the Neolithic population from the Marmara region in north-western Turkey. These results support the generally accepted direction of migrations, associated with the spread of the Neolithic. The idea that the spread of the Neolithic was connected with the direct migration of populations from the Near East through Anatolia and then through the Balkans and Mediterranean coastlines has been the dominant hypothesis among archaeologists [67,68] even before the aDNA studies provided direct evidence of this process [28,69,70,71]. This is further supported by both of those populations having a close affinity with Neolithic Greece and Macedonia, as seen in PCA and t-SNE results, as those regions have undergone the Neolithic Transition relatively early and were under the direct influence of north western Anatolia [27,72,73]. The role of Central Anatolia in this process was also supported by whole genome data from several individuals from Tepecik-Çiftlik and Boncuklu Höyük [30]. All of the data seems to favor the idea that the Neolithic in the Marmara Region emerged as a result of expansion, potentially originating in Central Anatolia [15,22,74].

Generally, the Central Anatolian Neolithic is thought to be unique and clearly distinguished from the Neolithic core zone in the Levant and northern Mesopotamia [27]. Our results show that the Central Anatolian Neolithic, while falling within the genomic diversity of Near and Middle Eastern Neolithic populations, is always set apart from the groups from the Fertile Crescent. This supports the idea of a major involvement of local populations in adopting the Neolithic lifestyle in Central Anatolia proposed by archeologists [16,17,18]. This potential genetic uniqueness of Central Anatolia was recently supported by whole genome data from Boncuklu Höyük [30], and is further supported by our findings showing that the set of samples from Çatalhöyük VIA level was also a part of this distinctive Central Anatolian population.

Despite the fact that the Neolithic in Central Anatolia might have been adopted by local hunter-gatherers [16,17] with no gene flow from the south eastern Anatolia and the Levant, it still might be hard to genetically distinguish various Anatolian populations from each other, as all of them share a similar upper-Paleolithic background. However, aDNA data from southern Levant and Zagros Mountains points towards large genetic differentiation of both early Neolithic populations and their direct upper Paleolithic predecessors [69]. This variability of the Epipaleolithic background, if we assume the independent adaptation of the Neolithic by local Central Anatolian hunter-gatherers, is also supported by our results, which show that Levantine and Middle Eastern populations do not cluster with groups from Anatolia.

## 5. Conclusions

Our data suggests a lack of maternal kinship among ten analyzed individuals buried under the floors of selected adjacent Çatalhöyük buildings. This result can be interpreted as a sign that those burials were representing either large kin group with multiple maternal lineages, or a group of individuals selected for burial based on foundations other than genetic affinity. This fits well with previous research based on morphological traits of the human remains from the site. Population analyses show that the Central Anatolian Population, including Çatalhöyük, falls within the genomic variability of Near and Middle East Neolithic, having the closest affinity to the population from the Marmara Region. This result supports the hypothesis about the direction of the spread of the Neolithics within the Anatolian Peninsula and beyond and emphasizes the significant role of Central Anatolia in this process.

## Figures and Tables

**Table 1:** Individuals for which the complete mitochondrial genomes were acquired in this study. n.a.: not available, Ry values in parentheses.

Skeleton Number	Building	Age at Death	Morphological Sex	Proportion of Human DNA (%)	Mt Coverage	Molecular Sex	Mitochondrial Haplogroup
20036	80	child (3–12)	n.a.	3.3	235.94	Probable XY (0.0729)	U3b
19159	80	adolescent (12–20)	n.a.	4.5	14.30	Probable XY (0.0783)	N
21981	89	infant (0–3)	n.a.	26.6	8.14	XX (0.0005)	K1a17
30900	89	infant (0–3)	n.a.	1.0	35.07	Probable XY (0.0665)	U
20810	96	adult (20+)	male?	0.5	21.34	Not assigned	W1c
19727	96	child (3–12)	n.a.	2.0	24.83	XY (0.107)	K
20832	96	older adult (50+)	female?	0.6	7.18	XX (0.0042)	H+73
20850	96	child (3–12)	n.a.	0.5	10.02	XX (0.0027)	H
20374	97	mature adult(35–50)	male?	1.3	41.40	Not assigned	X2b4
20351	97	adolescent (12–20)	female?	0.6	17.91	Not assigned	U5b2

## Supplementary Materials

The following are available online at https://www.mdpi.com/2073-4425/10/3/207/s1. Supplementary information SI (including Supplementary Text, Supplementary Figures S1–S5 and Supplementary Table S1), Supplementary_datasets_S1–S3.
