# Supplementary material for: Ancient Mitochondrial Genomes Reveal the Absence of Maternal Kinship in the Burials of Çatalhöyük People and Their Genetic Affinities
Source: Genes (Basel). 2019 Mar 11;10(3):207. doi: 10.3390/genes10030207 (PMC6471721; doi:10.3390/genes10030207)
Supplement: Supplementary file 1 [file genes-10-00207-s001.zip › Genes_Chylenski SI_2.docx]

Supplementary Information for

**Ancient mitochondrial genomes reveal the kinship structure and genetic affinities of Neolithic Çatalhöyük people**

Maciej Chyleński^1*^, Edvard Ehler^2^, Mehmet Somel^3^, Reyhan Yaka^3^, Maja Krzewińska^4^, Mirosława Dabert^5^, Anna Juras^6^, Arkadiusz Marciniak^1^

Maciej Chyleński^1^

Email: [maciej.ch@amuy.edu.pl](mailto:maciej.ch@amuy.edu.pl)

**This PDF file includes:**

Supplementary text

Figs. S1 to S5

Table S1

References for SI reference citations

**Other supplementary materials for this manuscript include the following:**

Datasets S1 to S3

# S1. Detailed archaeological context for the samples for which complete mitochondrial genomes were acquired.

**Sk. 20810**

The individual was found in the year 2013 within a primary burial (Feature 7003) found in central eastern platform of building 96. The skeleton was complete but poorly preserved and based on morphological traits it was interpreted as an adult (20+ years). Sex was recorded as probable male based on the characteristics of the cranium and mandible. The individual was interred in very tightly flexed position and was lying on its left side with the head to the west and feet to the east [1].

Lower right premolar was sampled for the aDNA analysis.


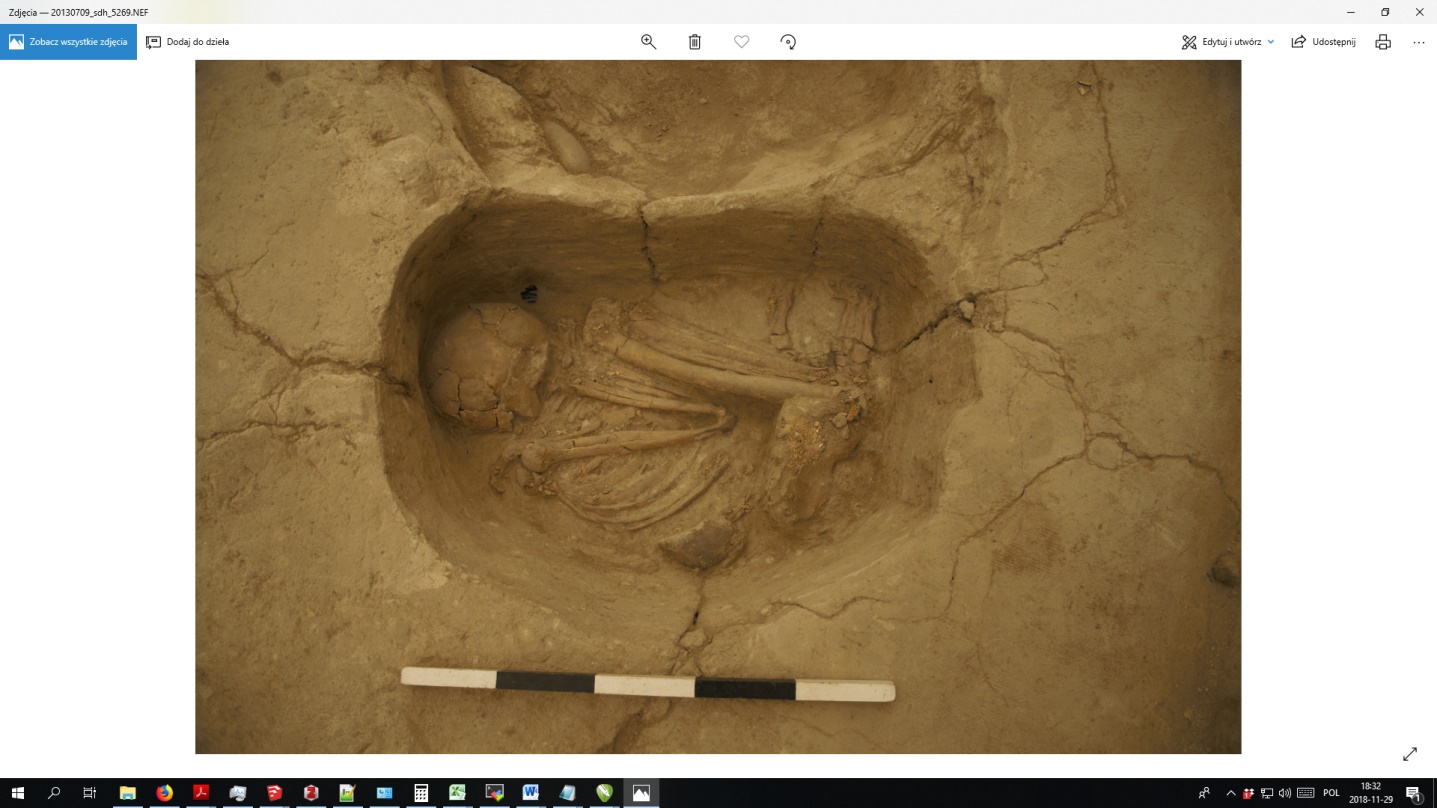
Skeleton 20810 in situ (photo Scott Haddow)

**Sk. 19727**

The individual was excavated in 2012 from the burial (feature 7002) in north eastern platform of building 96. The skeleton was a very tightly flexed juvenile, covered by a very clear layer of phytoliths, indicative of a mat or basket associated with the burial. The remains were quite well preserved and the individual was interpreted to be a child (3-12 year old at death), due to this the sex could not be defined based on morphological traits [2].

Petrous part of right temporal bone was sampled for the aDNA study.


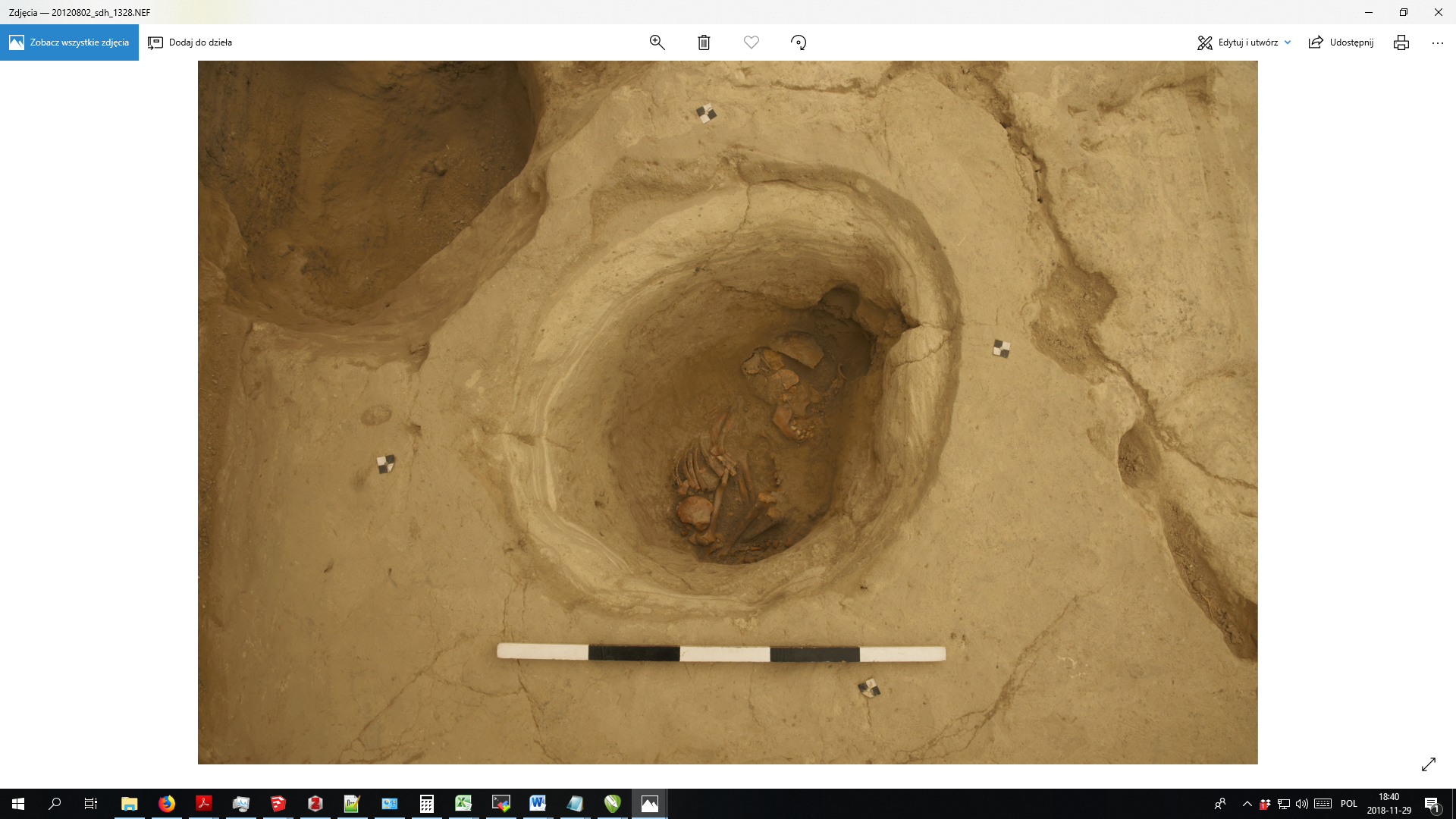


Skeleton 19727 in situ (photo Scott Haddow)

**Sk. 20832**

The individual was found during 2013 excavation season within primary burial (feature 7011) under southeastern platform of building 96. The relatively complete but poorly preserved skeleton was interpreted to belong to an adult female. The individual was interred in a tightly flexed position and lying on its right side. The cranium of another individual (Skeleton 20830), was placed in association with this individual [1]l.

Lower left canine was sampled for the aDNA study.


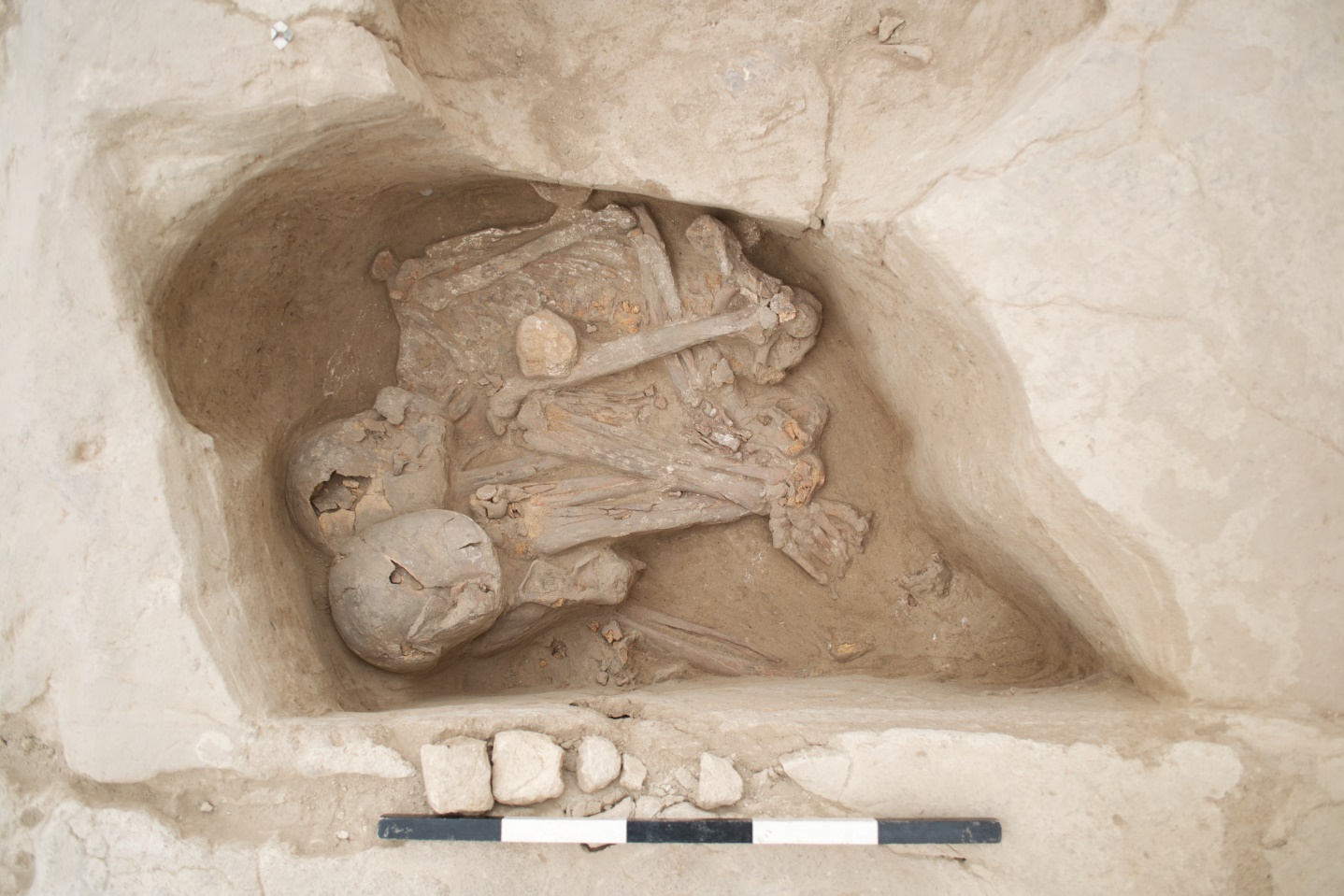
Skeleton 20832 (top) and cranium 20830 (bottom) (photo Johanna Bergqvist)

**Sk. 20850**

The remains of this individual were excavated in 2014 from a burial (feature 7012) under central eastern platform of building 96. The relatively poorly preserved remains were lying in a tightly flexed and supine position and were interpreted as belonging to a child (3-12 years old at death) [3].

Petrous part of right temporal bone was sampled for the aDNA analysis.


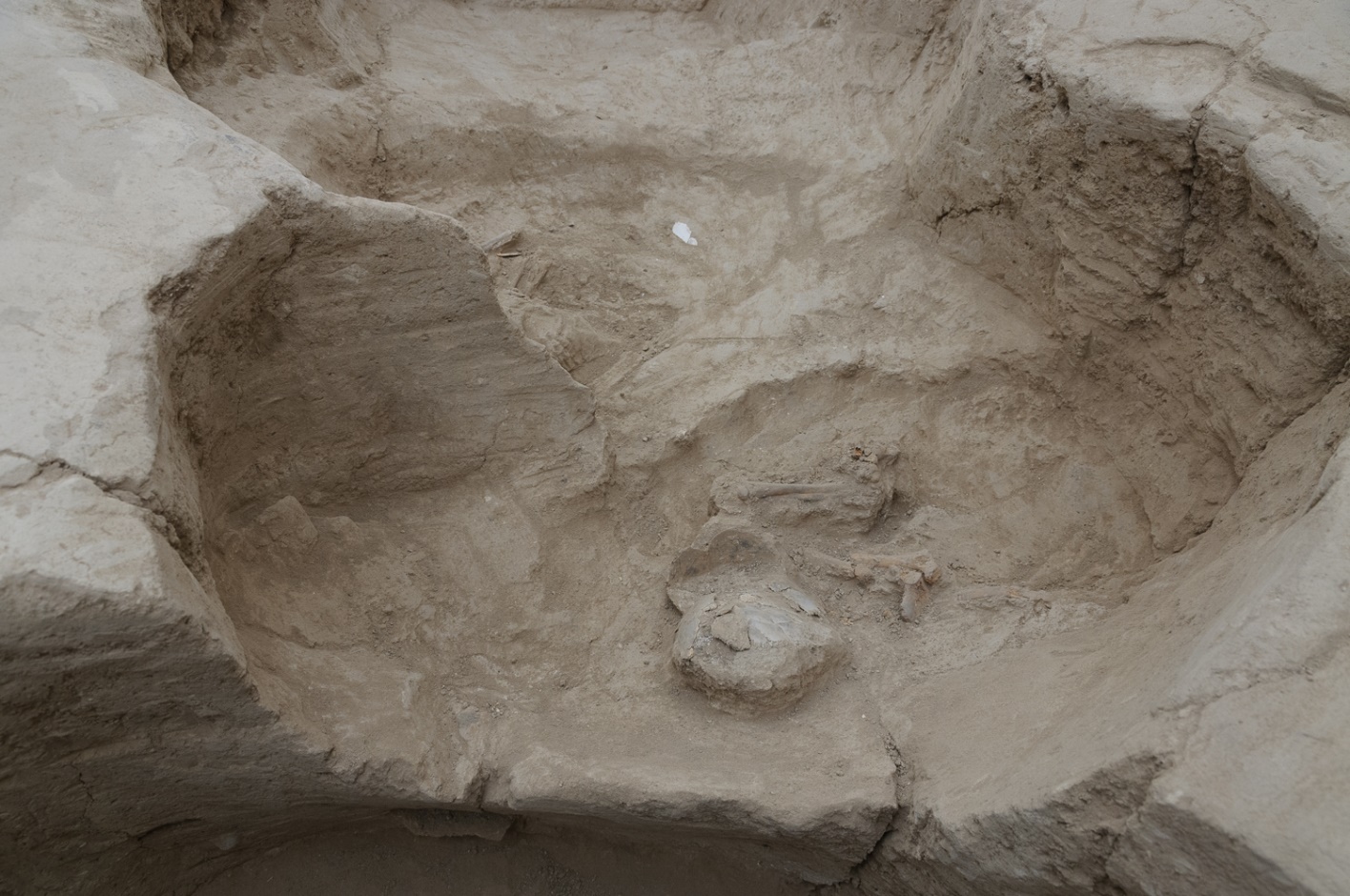
 Skeleton 20850 in situ (photo Scott Haddow).

**Sk. 20351**

The individual was found during 2012 excavation season in burial (feature 3552) under northern central platform of building 97. The individual was interpreted to be a late adolescent (12-20 year old) probable female. The skeleton was flexed and poorly preserved and disturbed by later burials from the sequence [2].

Upper left incisor and petrous part of temporal bone were sampled for the aDNA analysis.


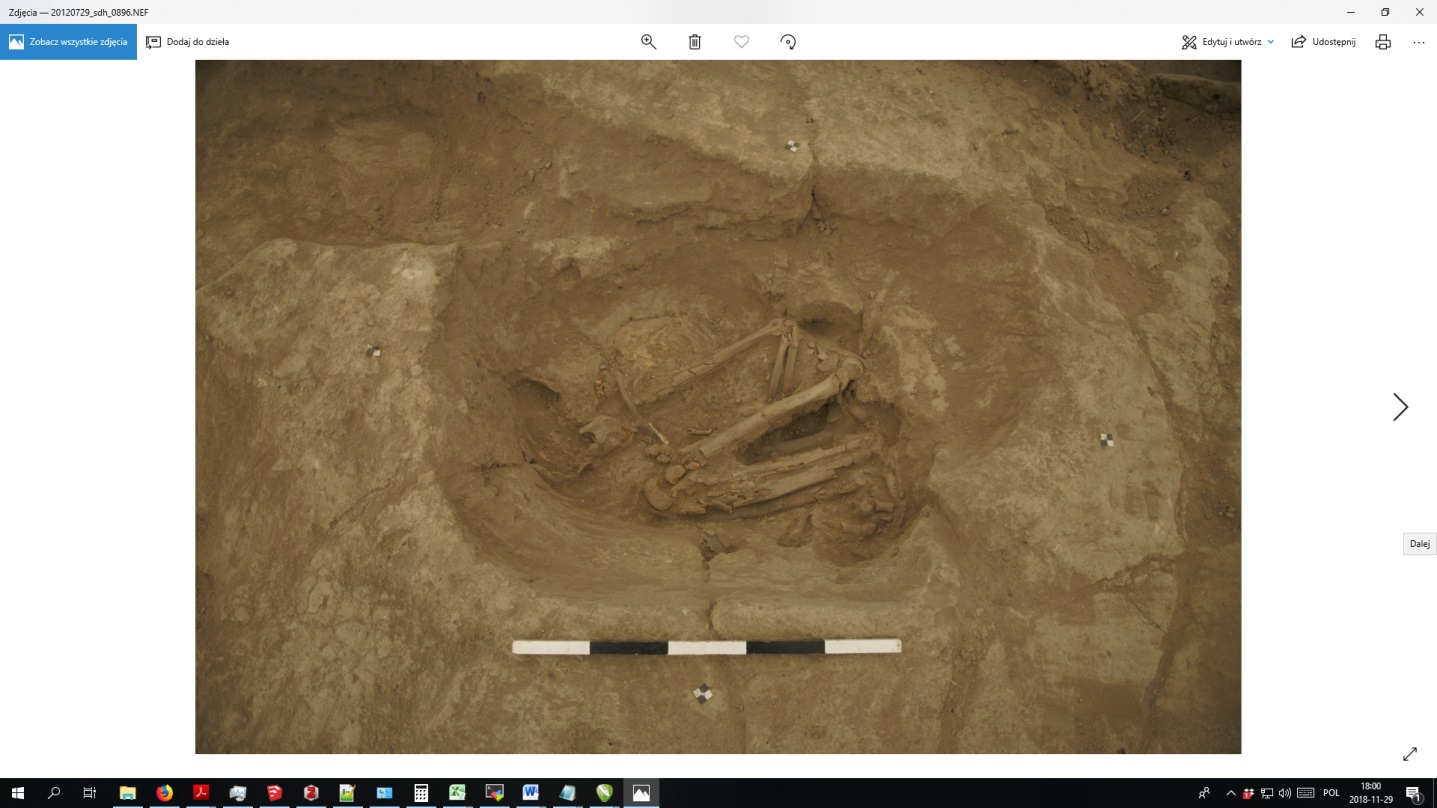


Skeleton 20351 in situ (photo Scott Haddow)

**Sk. 20374**

The individual was unearthed during 2012 excavation season in burial (feature 3555) within central northern platform of building 97. The burial was interpreted as primary disturbed burial of an adult (30-40 years) male. The skeleton was relatively complete and in good condition [2].

Upper left incisor was sampled for the aDNA study.


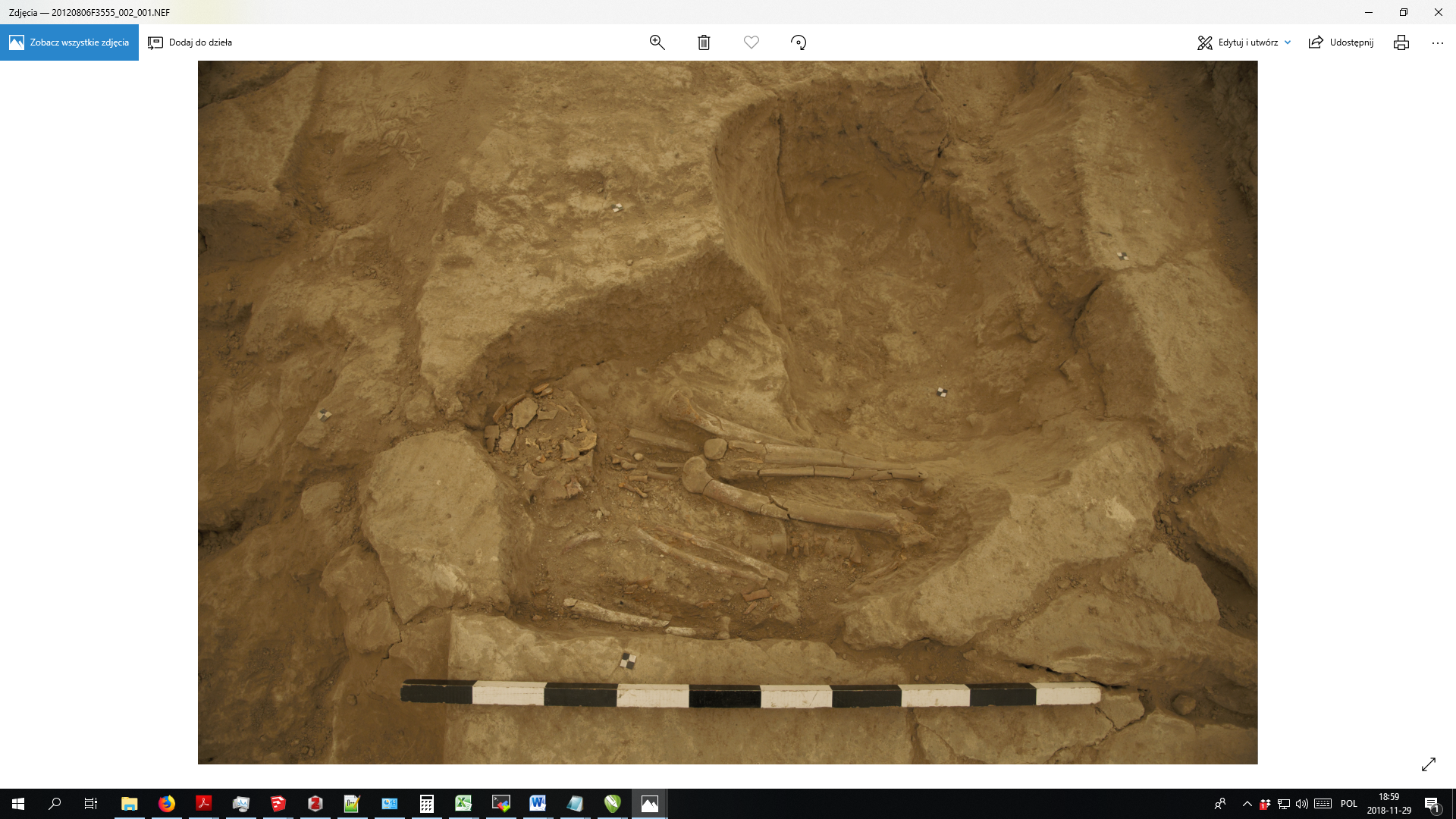
Skeleton 20374 in situ (photo Scott Haddow)

**Sk. 20036**

The remains of this individual were unearthed in 2013 in a burial (feature 7400) under the northeastern platform of building 80. The remains of several individuals were found in this burial, the number 20036 was given to disarticulated juvenile cranium laying in the northern part of the grave on its right side oriented with its apex in a northwesterly direction, facing southwest [1]. Later the cranium was interpreted to belong to one of the individuals found in earlier double burial (feature 7420) [4].

Petrous part of left temporal bone was sampled for the aDNA analysis.


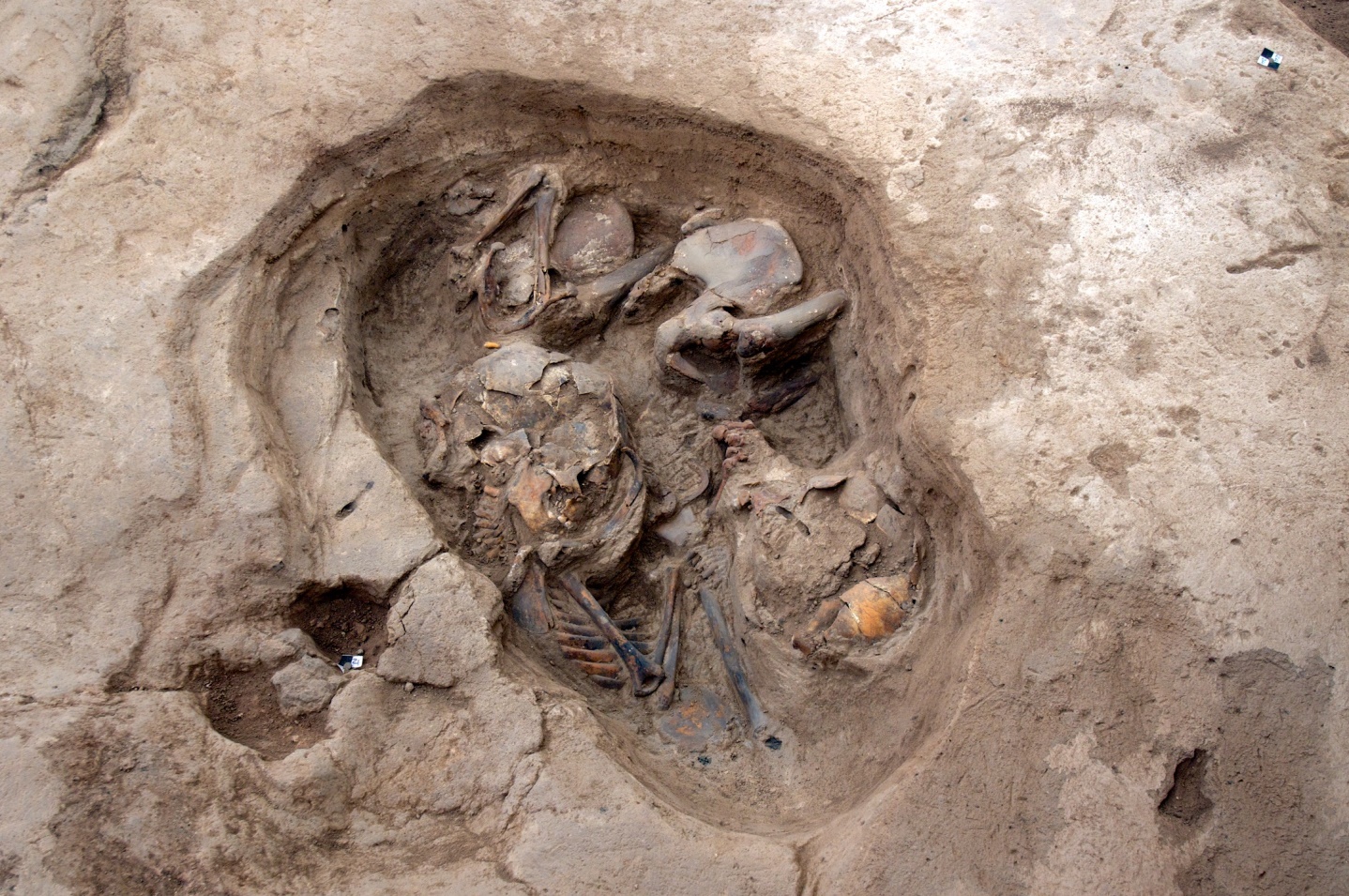
 Cranium 20036 (bottom right) next to skeleton 20034 (left) and 20039 (top) in situ (photo Scott Haddow)

**Sk. 19159**

The individual was found during 2016 excavation season in the primary disturbed double burial (feature 7420) under the northwest platform (F.3442) of building 80. The tightly flexed skeleton was interpreted as belonging to adolescent [4].

Petrous part of right temporal bone was sampled for the aDNA analysis.

 Skeleton 19159 in situ (photo Jason Quinlan).

**Sk. 21981**

The individual was found in burial (feature 8153) under the central northern platform of building 89. The remains of this infant were lying flexed on its left side and the lower part of the skeleton was truncated later during the production of a post retrieval pit. The tight flexion of the skeleton suggested some form of wrapping, confirmed by the presence of phytoliths in the region of the hands [5].

Petrous part of right temporal bone was sampled for the aDNA study.


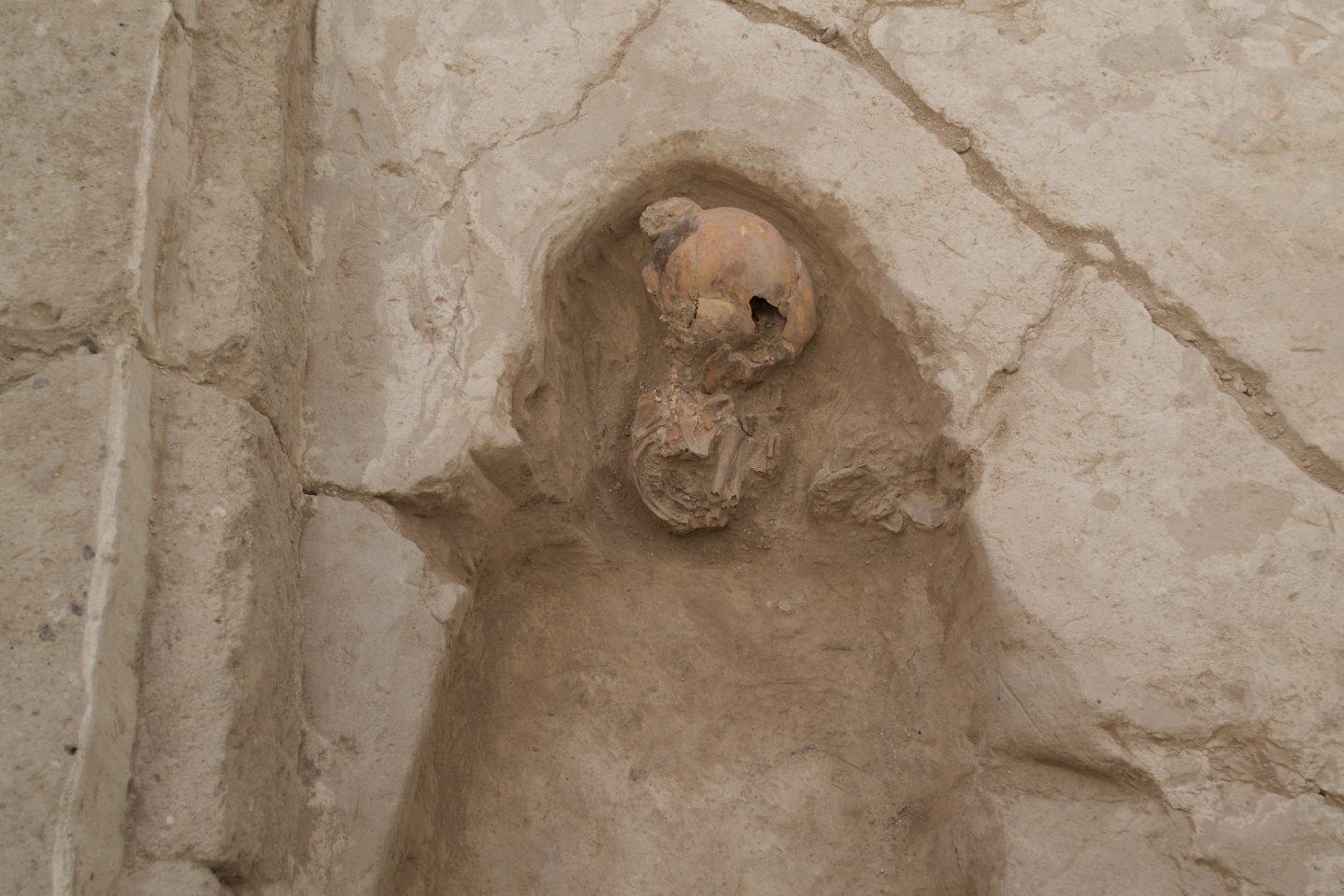
Skeleton Skeleton 21981 in situ (photo Jason Quinlan).

**Sk. 30900**

The individual was found during 2013 excavation season within primary burial (feature 3478). The relatively complete and well preserved skeleton belonged to a young subadult (infant) and was buried in a flexed position, lying on its left side [1].

Petrous part of left temporal bone was sampled for the aDNA analysis.


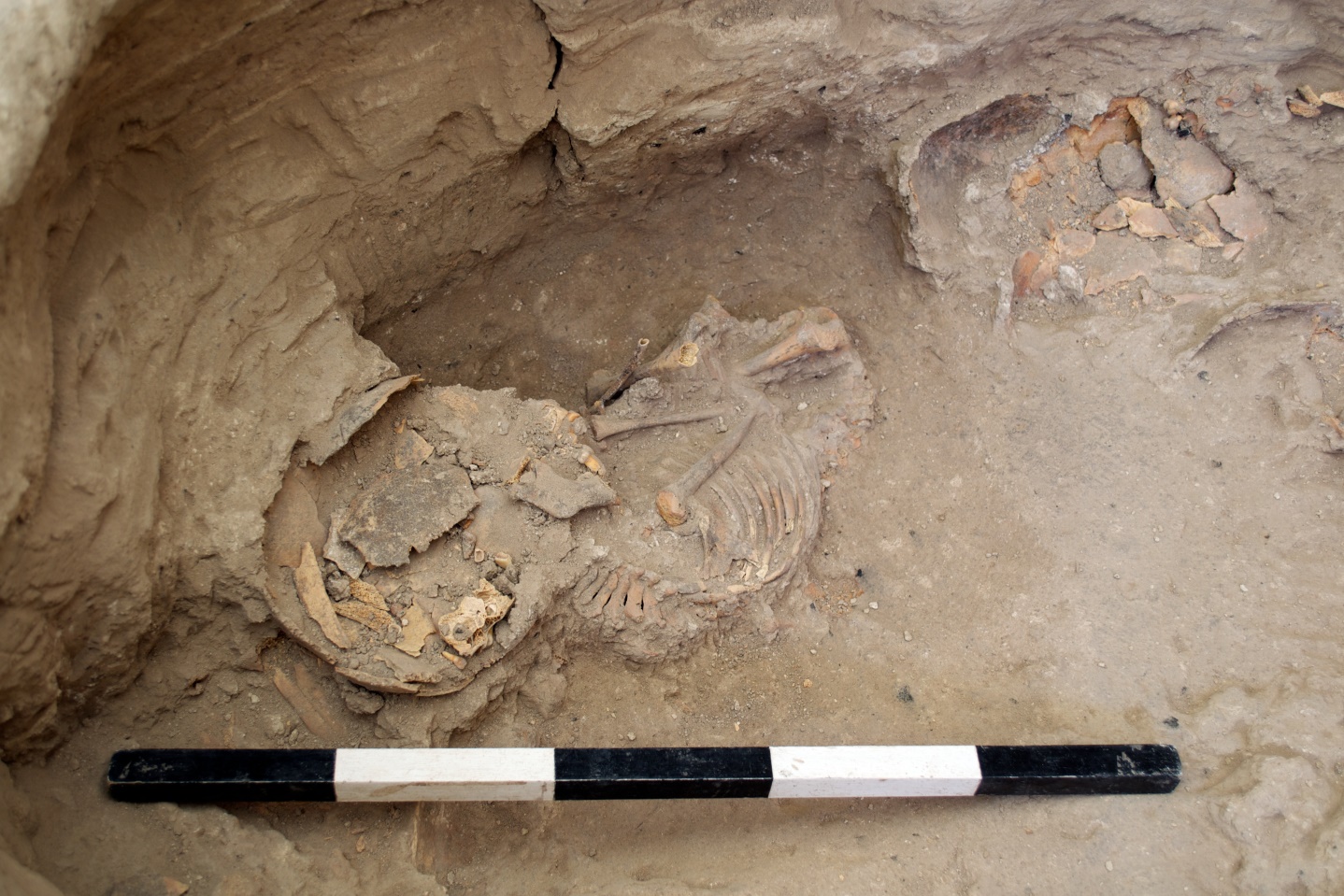
Skeleton 30900 in situ (photo Chiara Mottolese).

**
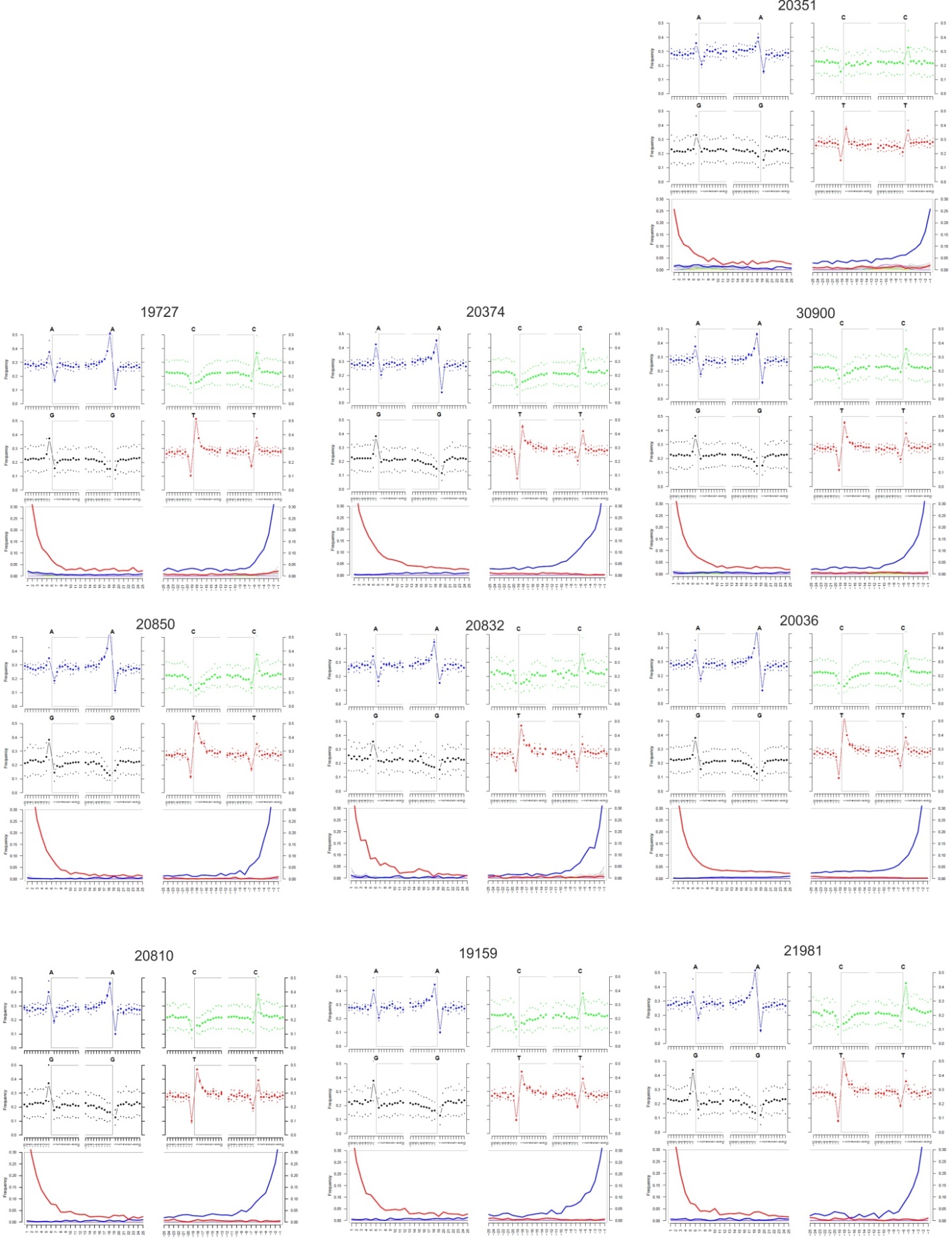
**

Fig. S1. The post mortem damage patterns obtained with the use of mapDamage v2.0.5.


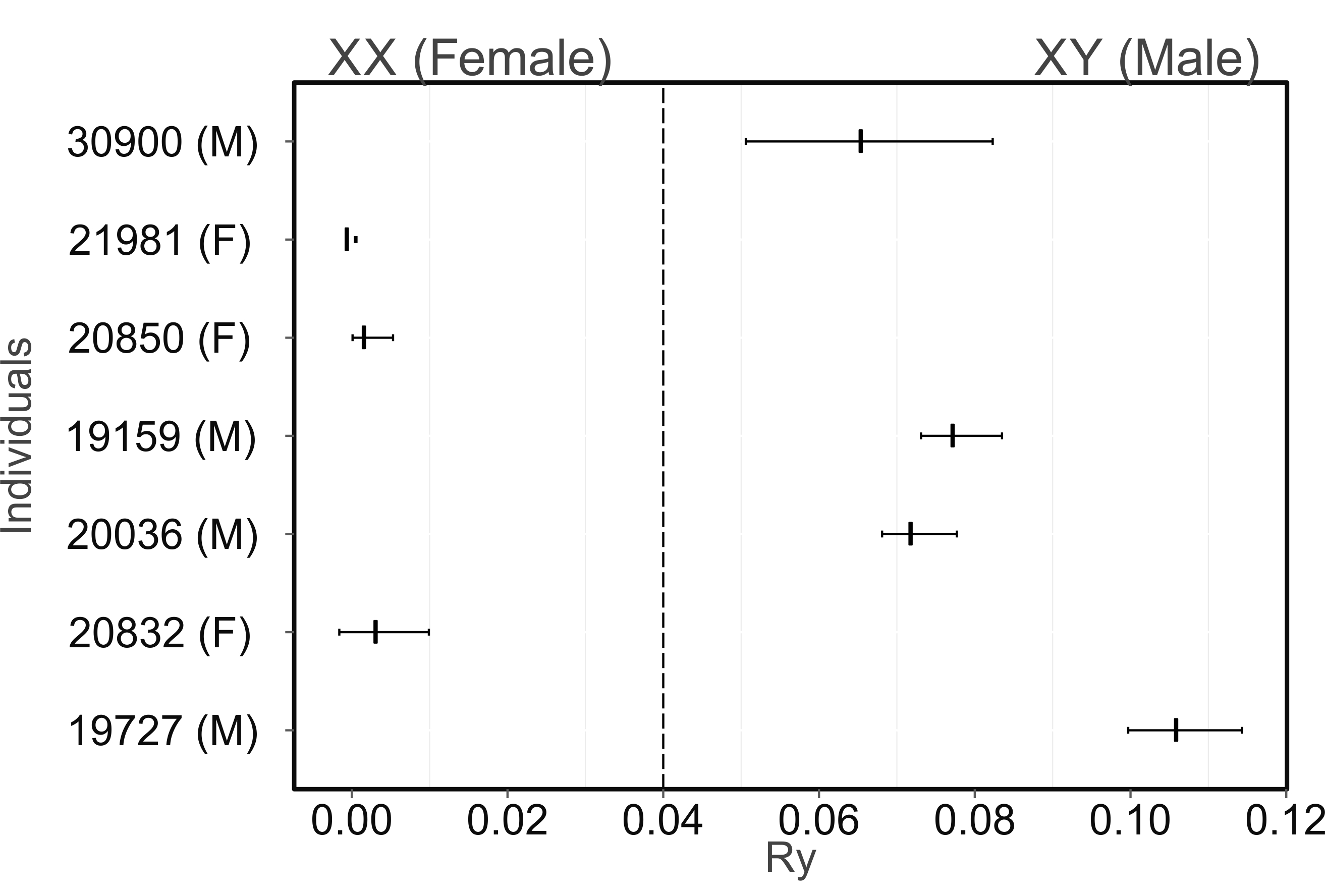


Fig. S2. The ratio of reads mapping to Y and X chromosomes (Ry) used for molecular sex assignment.


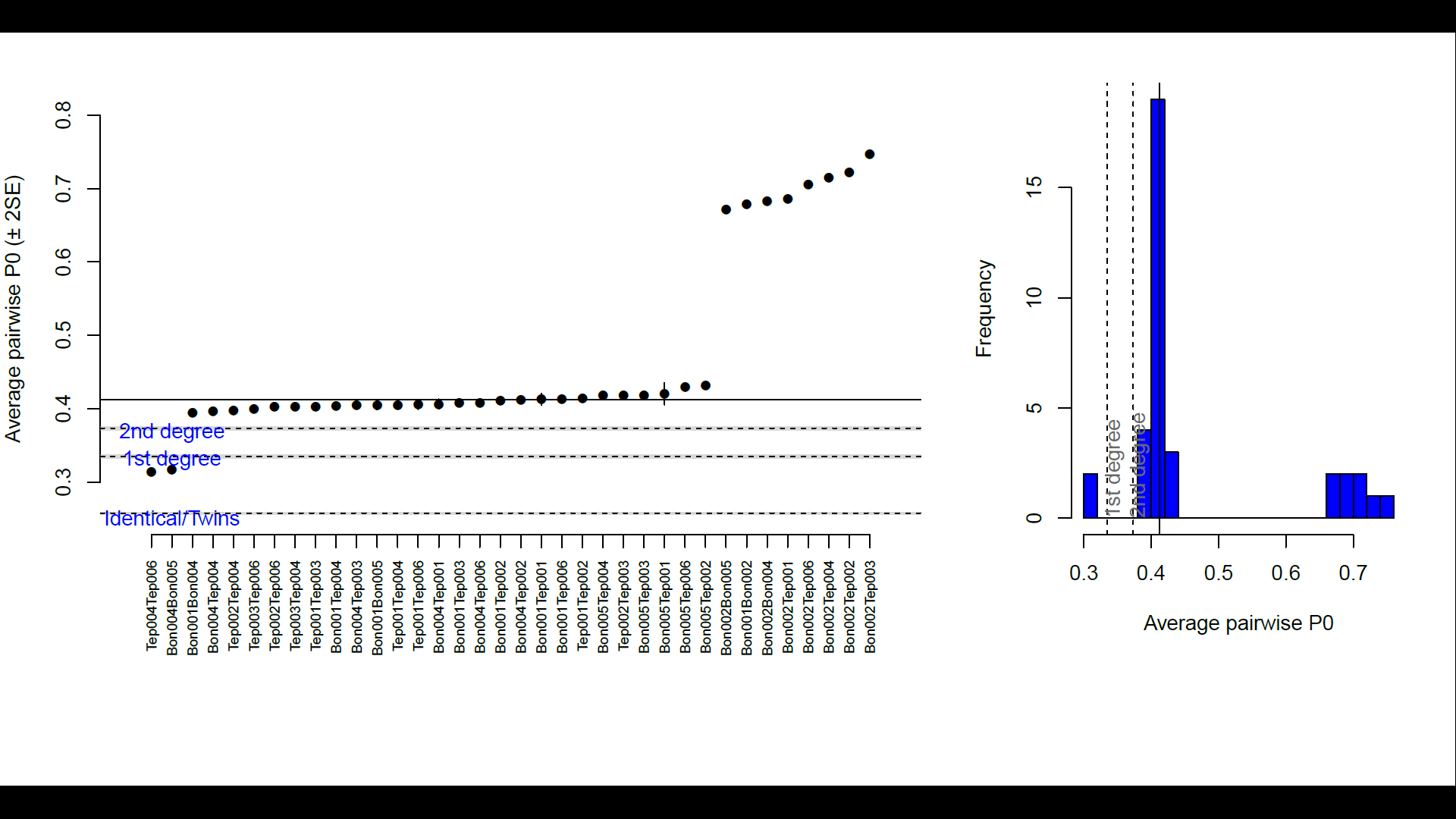


Fig. S3. Relatedness estimation of Central Anatolian Neolithic samples with the use of READ.


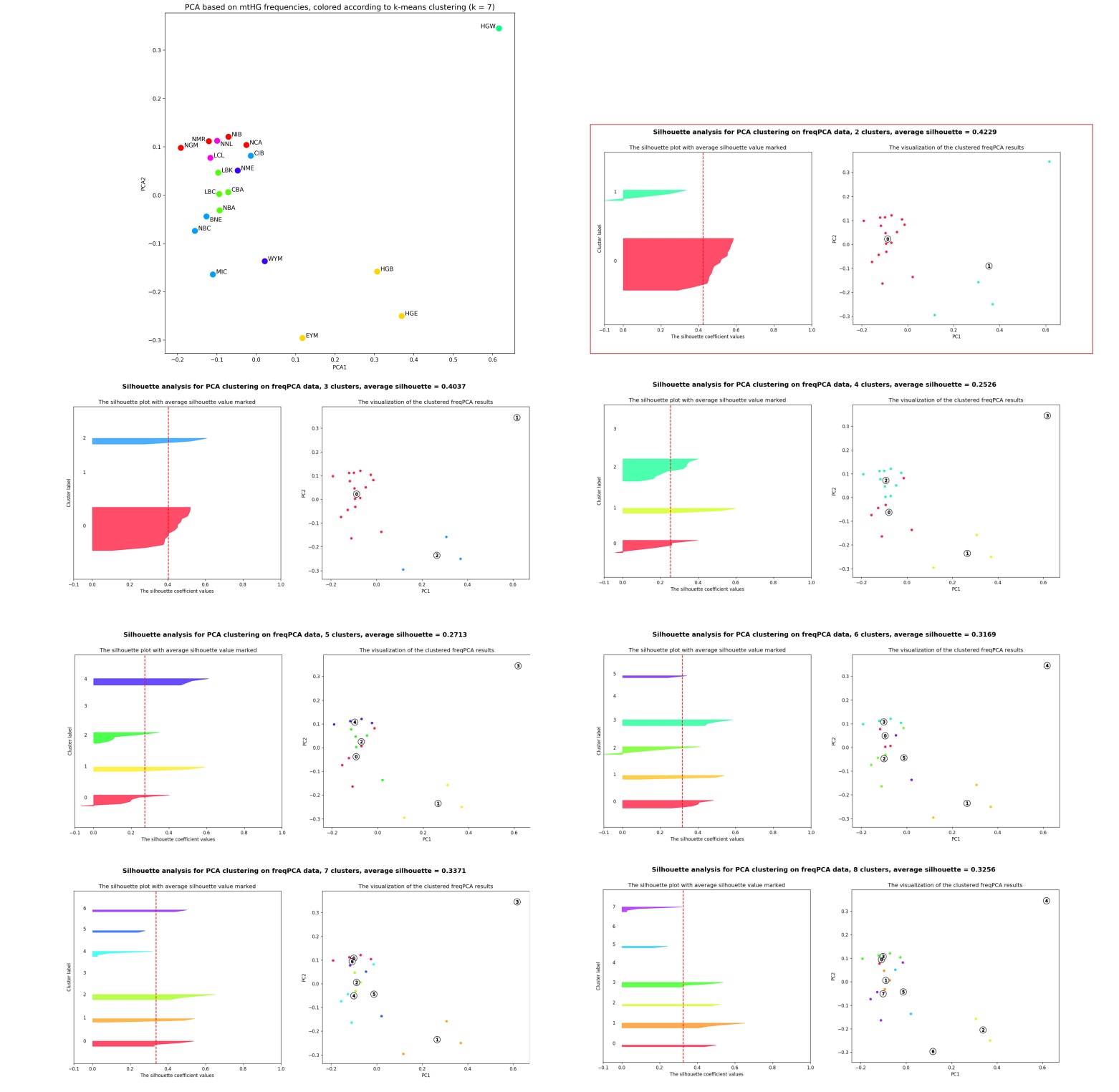
Fig. S4A. k-means variants (from 2 to 8) for PCA with average silhouette shown for extended dataset.


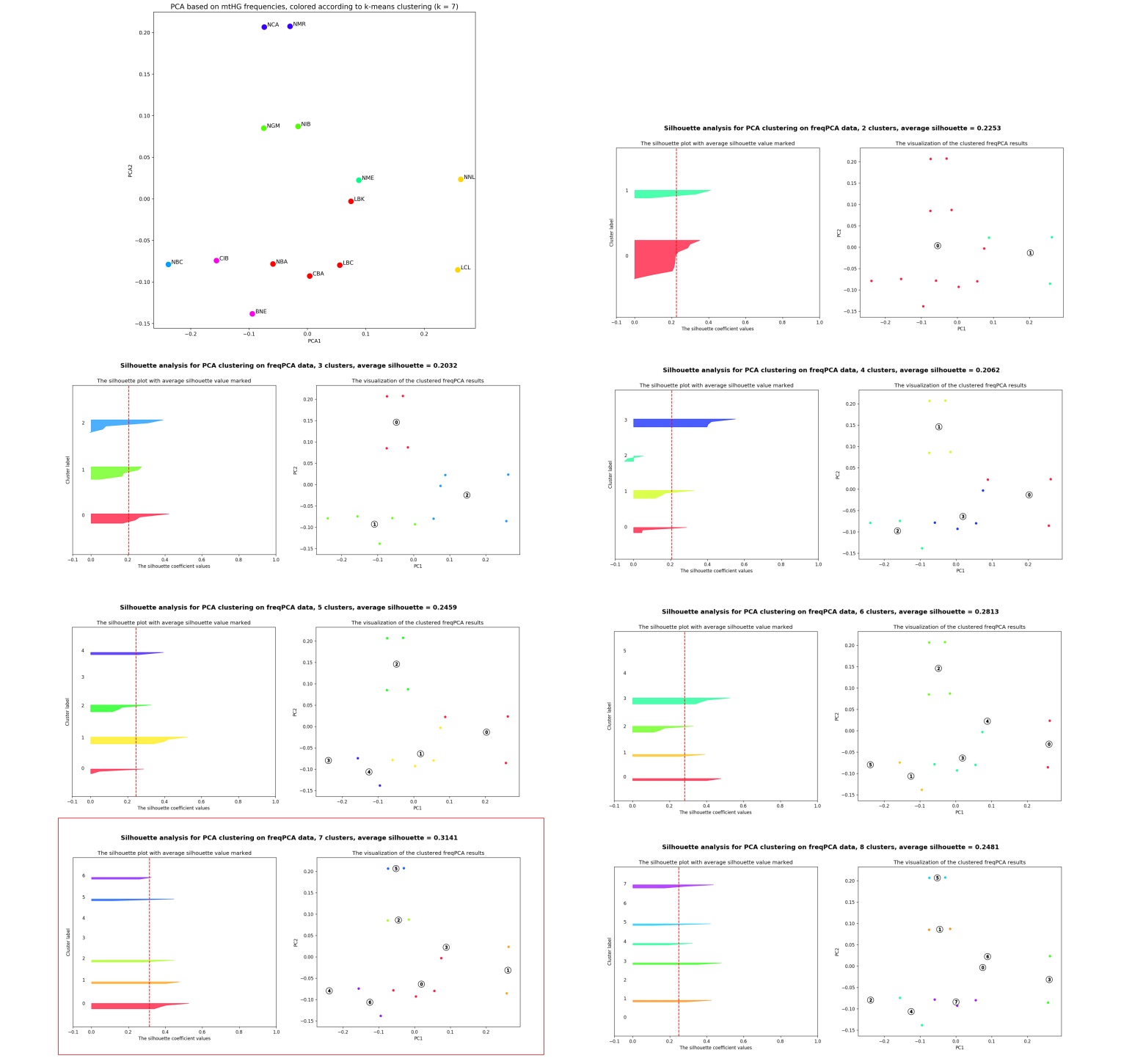


Fig. S4B. k-means variants (from 2 to 8) for PCA with average silhouette shown for reduced dataset.


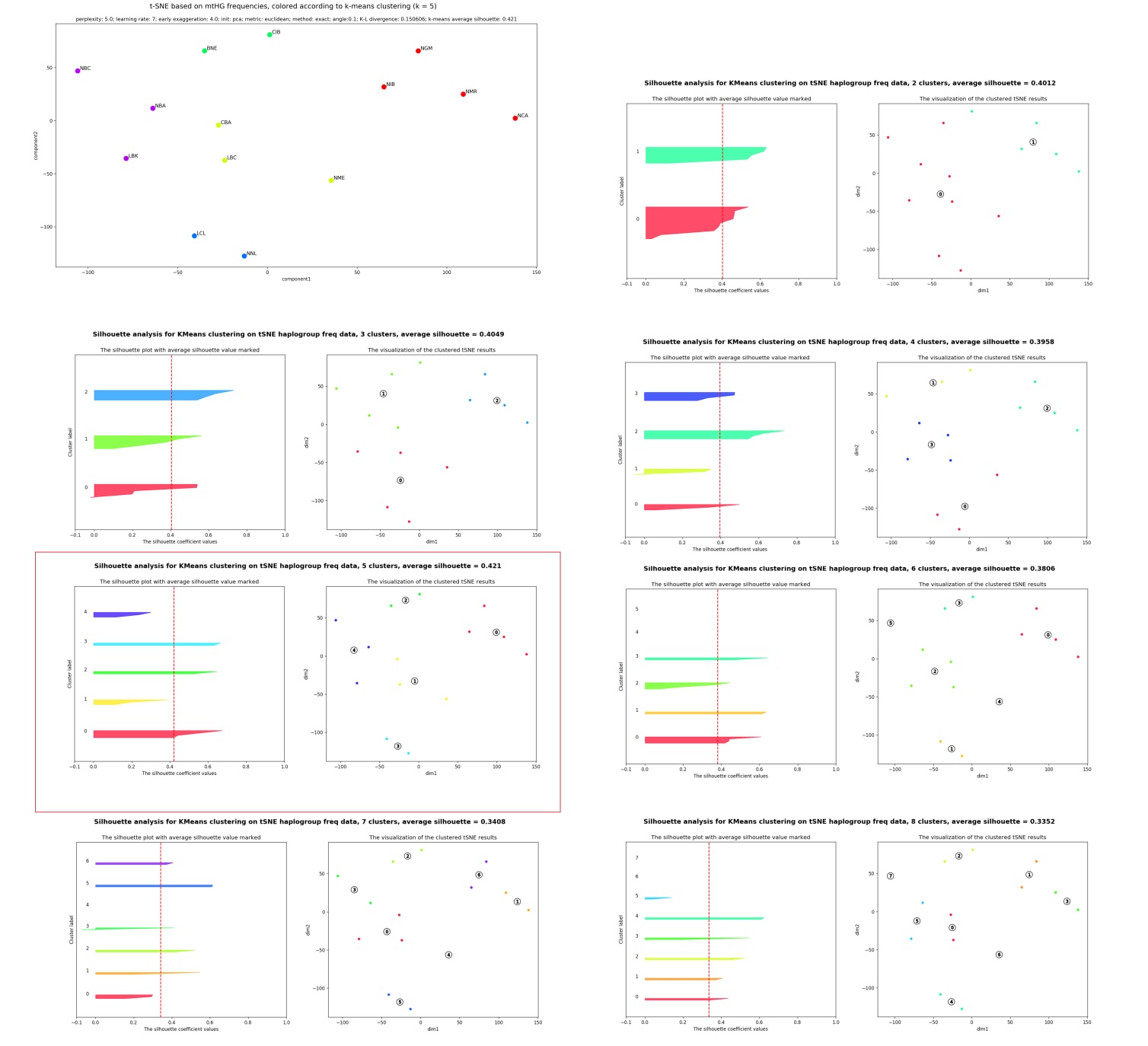


Fig. S4C. k-means variants (from 2 to 8) for t-SNE with average silhouette shown for reduced datasets.





Fig. S4D. k-means variants (from 2 to 8) for t-SNE with average silhouette shown for extended datasets.

Table S1. Relatedness estimation of Central Anatolian Neolithic samples with the use of READ.

| Pair of Individuals | Relationship | Z_upper | Z_lower | Overlapping SNPs (MAF>0.2) |
| --- | --- | --- | --- | --- |
| Bon001Bon002 | Unrelated | NA | -276.27615651 | 596946 |
| Bon001Bon004 | Unrelated | NA | -11.7067794269 | 202290 |
| Bon001Bon005 | Unrelated | NA | -9.71238001238 | 39437 |
| Bon001Tep001 | Unrelated | NA | -9.87998763507 | 24025 |
| Bon001Tep002 | Unrelated | NA | -26.582766259 | 395685 |
| Bon001Tep003 | Unrelated | NA | -23.5322966655 | 338834 |
| Bon001Tep004 | Unrelated | NA | -20.1724158173 | 309049 |
| Bon001Tep006 | Unrelated | NA | -23.2823607265 | 220516 |
| Bon002Bon004 | Unrelated | NA | -289.009369172 | 843238 |
| Bon002Bon005 | Unrelated | NA | -179.01403105 | 152107 |
| Bon002Tep001 | Unrelated | NA | -171.545748509 | 102736 |
| Bon002Tep002 | Unrelated | NA | -379.808000162 | 1832564 |
| Bon002Tep003 | Unrelated | NA | -430.941351009 | 1940899 |
| Bon002Tep004 | Unrelated | NA | -383.145323299 | 1421189 |
| Bon002Tep006 | Unrelated | NA | -363.543231893 | 955823 |
| Bon004Bon005 | First Degree | 6.15781879755 | -20.7795480687 | 53792 |
| Bon004Tep001 | Unrelated | NA | -9.83142213319 | 32746 |
| Bon004Tep002 | Unrelated | NA | -28.8092260446 | 557420 |
| Bon004Tep003 | Unrelated | NA | -21.9887027225 | 479698 |
| Bon004Tep004 | Unrelated | NA | -16.3380722603 | 435711 |
| Bon004Tep006 | Unrelated | NA | -22.7489823202 | 310926 |
| Bon005Tep001 | Unrelated | NA | -6.29897992378 | 6707 |
| Bon005Tep002 | Unrelated | NA | -28.3579308058 | 101523 |
| Bon005Tep003 | Unrelated | NA | -20.705833938 | 87083 |
| Bon005Tep004 | Unrelated | NA | -19.4694926526 | 80093 |
| Bon005Tep006 | Unrelated | NA | -20.9630103023 | 58265 |
| Tep001Tep002 | Unrelated | NA | -16.8397922416 | 64627 |
| Tep001Tep003 | Unrelated | NA | -11.9490684803 | 58757 |
| Tep001Tep004 | Unrelated | NA | -11.477775769 | 49785 |
| Tep001Tep006 | Unrelated | NA | -10.1146797761 | 36970 |
| Tep002Tep003 | Unrelated | NA | -36.896783433 | 1858470 |
| Tep002Tep004 | Unrelated | NA | -19.9792727567 | 1057484 |
| Tep002Tep006 | Unrelated | NA | -22.231516104 | 889323 |
| Tep003Tep004 | Unrelated | NA | -24.1709526833 | 622174 |
| Tep003Tep006 | Unrelated | NA | -19.1443427887 | 846566 |
| Tep004Tep006 | First Degree | 17.0097539783 | -45.0779942996 | 545124 |

Additional data table S1 (separate file)

**S1. Statistics of the shotgun and capture libraries sequenced for the study**

**S2. Detailed information on the samples used for population genetics analyses**

**S3. Linearized FST distances based on complete mtDNA sequences.**

**References**

[1] J. Tylor, Excavations in the South Area, in: B. Tung (Ed.), Çatalhöük 2013 Arch. Rep., 2013: pp. 45–73. http://www.catalhoyuk.com/sites/default/files/media/pdf/Archive_Report_2013.pdf.

[2] J. Tylor, Excavations in the South Area, in: B. Tung (Ed.), Çatalhöük 2012 Arch. Rep., 2012: pp. 35–61. http://www.catalhoyuk.com/sites/default/files/media/pdf/Archive_Report_2012.pdf.

[3] J. Tylor, Excavations in the South Area, in: H. Scott (Ed.), Çatalhöük 2014 Arch. Rep., 2014: pp. 43–71. http://www.catalhoyuk.com/sites/default/files/media/pdf/Archive_Report_2014.pdf.

[4] J. Tylor, Excavations in the South Area, in: H. Scott (Ed.), Çatalhöük 2016 Arch. Rep., 2016: pp. 51–72. http://www.catalhoyuk.com/sites/default/files/media/pdf/Archive_Report_2016.pdf.

[5] J. Tylor, Excavations in the South Area, in: H. Scott (Ed.), Çatalhöük 2015 Arch. Rep., 2015: pp. 43–71. http://www.catalhoyuk.com/sites/default/files/media/pdf/Archive_Report_2015.pdf.
